# Excreted/secreted *Schistosoma mansoni* venom allergen-like 9 (SmVAL9) modulates host extracellular matrix remodelling gene expression

**DOI:** 10.1016/j.ijpara.2014.04.002

**Published:** 2014-07

**Authors:** Timothy P. Yoshino, Martha Brown, Xiao-Jun Wu, Colin J. Jackson, Ramon Ocadiz-Ruiz, Iain W. Chalmers, Marlen Kolb, Cornelis H. Hokke, Karl F. Hoffmann

**Affiliations:** a Department of Pathobiological Sciences, School of Veterinary Medicine, University of Wisconsin, Madison, WI 53706, USA; b Institute of Biological, Environmental and Rural Sciences (IBERS), Aberystwyth University, Room 3.31, Edward Llwyd Building, Penglais Campus, Aberystwyth SY23 3DA, UK; c Department of Parasitology, Leiden University Medical Center, P.O. Box 9600, 2300 RC Leiden, The Netherlands

**Keywords:** *Schistosoma mansoni*, *Biomphalaria glabrata*, Matrix metalloproteinase, Venom allergen-like

## Abstract

The *Schistosoma mansoni* venom allergen-like (SmVAL) protein family consists of 29 members, each possessing a conserved α-β-α sandwich tertiary feature called the Sperm-coating protein/Tpx-1/Ag5/PR-1/Sc7 (SCP/TAPS) domain. While the SmVALs have been found in both excretory/secretory (E/S) products and in intra/sub-tegumental (non-E/S) fractions, the role(s) of this family in host/parasite relationships or schistosome developmental processes remains poorly resolved. In order to begin quantifying SmVAL functional diversity or redundancy, dissecting the specific activity (ies) of individual family members is necessary. Towards this end, we present the characterisation of SmVAL9; a protein previously found enriched in both miracidia/sporocyst larval transformation proteins and in egg secretions. While our study confirms that SmVAL9 is indeed found in soluble egg products and miracidia/sporocyst larval transformation proteins, we find it to be maximally transcribed/translated in miracidia and subsequently down-regulated during in vitro sporocyst development. SmVAL9 localisation within sporocysts appears concentrated in parenchymal cells/vesicles as well as associated with larval germinal cells. Furthermore, we demonstrate that egg-derived SmVAL9 carries an N-linked glycan containing a schistosome-specific difucosyl element and is an immunogenic target during chronic murine schistosomiasis. Finally, we demonstrate that recombinant SmVAL9 affects the expression of extracellular matrix, remodelling matrix metalloproteinase (MMP) and tissue inhibitors of metalloproteinase (TIMP) gene products in both *Biomphalaria glabrata* embryonic cell (BgMMP1) and *Mus musculus* bone marrow-derived macrophage (MmMMP2, MmMMP9, MmMMP12, MmMMP13, MmMMP14, MmMMP28, TIMP1 and TIMP2) in vitro cultures. These findings importantly suggest that excreted/secreted SmVAL9 participates in tissue reorganisation/extracellular matrix remodelling during intra-mammalian egg translocation, miracidia infection and intra-molluscan sporocyst development/migration.

## Highlights

• *Schistosoma mansoni* VAL9 (SmVAL9) is a secreted N-linked glycoprotein containing a unique, difucosyl modification.
• SmVAL9 is found throughout miracidia/sporocyst parenchymal cell inclusions/vesicles and germinal cells.
• SmVAL9 differentially regulates murine and snail matrix metalloproteinases.

## Graphical abstract

**Figure:**
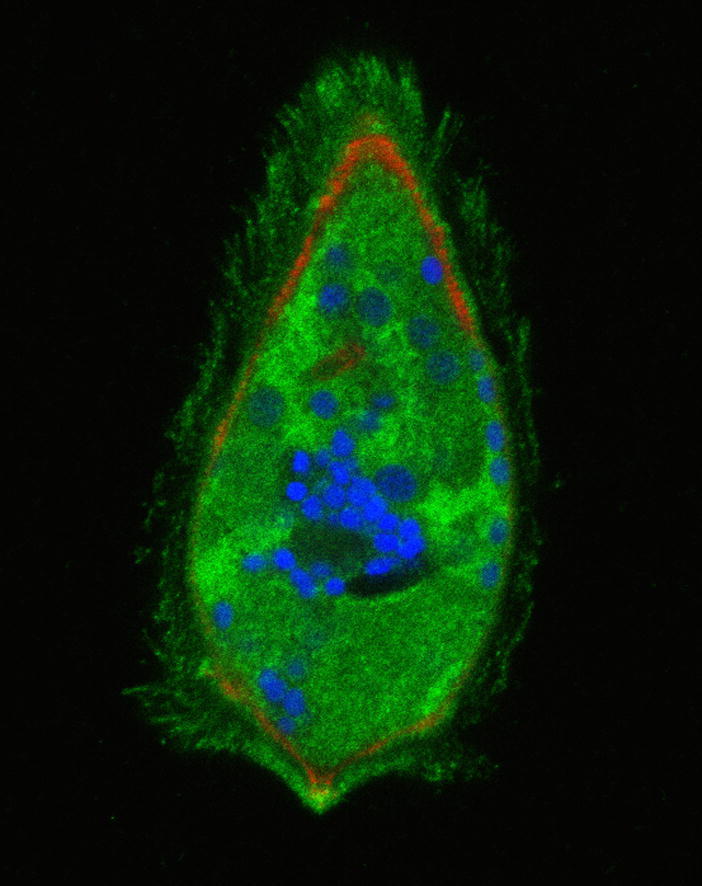

## 1 Introduction

It has long been appreciated that schistosomes are capable of establishing long-lasting relationships with their intermediate snail and definitive mammalian hosts (Basch, 1991). While the molecular basis for these parasite/host interactions is not fully understood (Geyer and Hoffmann, 2012), a variety of schistosome biomolecules including glycans (Hokke and Deelder, 2001; van Die and Cummings, 2010), proteins (Han et al., 2009), small metabolites (Da’dara and Skelly, 2011) and even microRNAs (miRNAs) (Cheng et al., 2013) are postulated to be involved. As schistosomiasis represents an important neglected tropical disease (NTD) targeted by international agencies for global elimination (Barry et al., 2013), identification and functional characterisation of the specific biomolecules utilised by schistosomes to orchestrate sustainable host interactions represents a rational approach in progressing novel chemotherapeutic/immunoprophylactic intervention strategies.

Recent studies in our laboratories have identified a family of proteins, the *Schistosoma mansoni* Venom Allergen-Like (SmVAL) molecules, which may be involved in parasite development and host interrelationships (Chalmers et al., 2008; Wu et al., 2009). The SmVALs are comprised of at least 29 members (SmVAL1–29) and are subdivided into two major groupings: the group 1 SmVALs (SmVAL1–5, 7–10, 12, 14–15, 18–29) and the group 2 SmVALs (SmVAL6, 11, 13, 16–17). Group 1 SmVALs display features (signal peptides and conserved cysteines appropriately positioned for disulphide bond formation) associated with an extracellular environment and excretion/secretion from the parasite whereas group 2 SmVALs do not (Chalmers et al., 2008). Interestingly, this segregation is not unique to schistosomes as group 1 and group 2 SmVAL homologs have also been identified in representative species across all four platyhelminth classes (Chalmers and Hoffmann, 2012). While basic information (localisation of transcript/protein to sub-surface tissues) related to group 2 SmVAL biology is limited to SmVAL6 (van Balkom et al., 2005; Nawaratna et al., 2011; Rofatto et al., 2012), experimental evidence to support the excretion/secretion of group 1 SmVALs from schistosomes is substantial. This includes the identification of SmVAL4, 10 and 18 in cercarial/schistosomula secretions (Curwen et al., 2006; Farias et al., 2012), SmVAL2, 3, 5 and 9 in egg secretions (Cass et al., 2007), SmVAL2, 3/23, 5/15, 9, 26/28, 27 and 29 from miracidial/sporocyst secretions (Wu et al., 2009), SmVAL26/28 from egg hatching fluid/secretions (Mathieson and Wilson, 2010; Farias et al., 2012) and SmVAL4 from cercarial infection tunnels (Hansell et al., 2008). Despite these reports confirming the presence of group 1 SmVALs at the host/parasite interface, no study has yet indicated a functional role for these proteins in establishing or maintaining schistosomiasis.

Towards this end, we believe that we provide the first experimental evidence that illustrates how an excreted/secreted (E/S) group 1 SmVAL (SmVAL9) influences host cell gene expression. While our data confirm that SmVAL9 is indeed secreted during miracidia to sporocyst transformation (likely from parenchymal or perikarya cells), we also show that this particular group 1 family member is decorated by a schistosome-specific double fucose-containing glycan in eggs and is immunogenic during murine schistosomiasis. These lifecycle expression patterns have been used to guide host cellular studies, which demonstrate that SmVAL9 affects the expression of extracellular matrix modifying gene products (metalloproteinases and tissue inhibitors of metalloproteinases) in both *Biomphalaria glabrata* embryonic (Bge) cell and *Mus musculus* bone marrow-derived macrophage (BMDMϕ) populations. This conserved activity affecting both snail and mammalian cells suggests that one function of SmVAL9 may be related to extracellular matrix remodelling, which is fundamentally important to schistosome lifecycle events including egg translocation, miracidia infection and sporocyst development. Further studies are necessary to understand whether this activity is an evolutionarily conserved function for all excreted/secreted group 1 SmVALs or is specific to egg/miracidia/sporocyst-derived SmVAL9.

## 2 Materials and methods

### 2.1 Ethics statement

All procedures performed on mice adhered to the United Kingdom Home Office Animals (Scientific Procedures) Act of 1986 as well as the European Union Animals Directive 2010/63/EU and were approved by Aberystwyth University’s (AU), UK ethical review panel and the University of Wisconsin-Madison’s (UW-Madison), USA Institutional Animal Care and Use Committee (IACUC, assurance number A3368-01).

### 2.2 Parasite material

Puerto Rican (AU and Leiden University Medical Center (LUMC), Netherlands) and NMRI (UW-Madison; University of Wisconsin, USA) strains of *S. mansoni* were used in this study. Cercariae were shed from *B.* *glabrata* intermediate snail hosts and used to percutaneously infect C57BL/6 mice (25 parasites/mouse) as previously described (Truscott et al., 2013). Miracidia were hatched from eggs harvested from mouse livers 7 weeks p.i., axenically isolated and placed into in vitro culture (Yoshino and Laursen, 1995). In vitro transforming miracidia and sporocyst stages were maintained for different times in culture (0.5 h up to 10 days) in Chernin’s balanced salt solution (Chernin, 1963) containing 1% PenStrep (HyClone, Thermo Scientific, USA) and 0.1% glucose/trehalose (MP Biomedicals, LLC, Solon, USA) (CBSS+) in 24-well tissue culture plates. Soluble egg antigens (SEAs) were prepared as previously described (Robijn et al., 2007). Equal numbers of miracidia, transforming sporocysts (0.5–8 h in culture) and fully transformed sporocysts (>24 h in culture) were removed from culture wells, rinsed 5x in CBSS + followed by 1x in snail PBS (sPBS, pH 7.2) and solubilised in SDS sample buffer (Novagen, EMD Millipore Corporation, USA). These particular samples were subjected to one freeze/thaw/vortex cycle, heated to 95 °C for 5 min, centrifuged at 16,000 *g* for 2 min and stored at −80 °C until needed. Media (CBSS+) from 24 h miracidium/sporocyst cultures containing larval transformation proteins (LTPs) and ciliated epidermal plates (EPs) shed into medium at 24 h were also obtained as previously described (Peterson et al., 2009; Wu et al., 2009). All soluble parasite protein samples were quantified by either a bicinchoninic acid assay (BCA, Thermo Scientific) or Bradford assay (Sigma–Aldrich, USA).

### 2.3 SmVAL9 transcription profile

Data from the 37,632 element *S. mansoni* long-oligonucleotide DNA microarray studies of Fitzpatrick et al. (2009) was interrogated to find the expression profile of SmVAL9 across 15 different lifecycle stages. Raw and normalised fluorescent intensity values are available via Array Express (https://www.ebi.ac.uk/arrayexpress/) under the experimental accession number E-MEXP-2094.

### 2.4 Recombinant SmVAL9 expression in *Escherichia coli*

The full-length SmVAL9 open reading frame (minus signal peptide) was amplified (forward 5′-TCT AGA ATG AAA ATG AAT GAC ACG ATT CGT G-3′; reverse 5′-CTC GAG TGC AGT CCT ATA CGG TCT TTG TTC-3′) from miracidium-derived cDNA (Chalmers et al., 2008) and cloned into the pGEM-T Easy vector system (Promega, UK). SmVAL9 inserts were excised by digestion with *Xba*I/*Xho*I and sub-cloned with a C-terminal 6 X histidine tag into the *E.* *coli* expression vector pET-30a (+) (Novagen; Merck Chemicals Ltd., UK).

The SmVAL9/pET-30a (+) construct was transformed into chemically competent *E. coli* One Shot BL21 Star (DE3) cells (Invitrogen, UK) and expression of recombinant (r)SmVAL9 carried out according to the BL21 Star instruction protocol. Induced cells were pelleted, lysed and centrifuged at 30,000*g* for 20 min. The resulting rSmVAL9 enriched inclusion bodies (IBs) were purified by first resuspending the cell pellet in wash buffer (50 mM Tris–HCl pH 8.0; 100 mM NaCl, 10 mM EDTA pH 8.0, 0.5% Triton X-100, 1.4 mM β-mercaptoethanol), followed by gentle agitation at 4 °C for 15 min. The IBs were repelleted and washed twice more as above in buffer containing 3 M urea. Purification of rSmVAL9 was carried out by size fractionation using a continuous elution electrophoresis apparatus (Model 491 Prep Cell; Bio-Rad, UK), following the manufacturer’s instructions. Fractions containing purified rSmVAL9 were pooled and concentrated in a 30 kDa MWCO centrifugal filter device (Amicon, Millipore, UK) to a final volume of 1 ml. Protein concentration was determined by the Bradford method (Sigma–Aldrich, UK) and aliquots of rSmVAL9 were stored at -80 °C.

### 2.5 Second stage rSmVAL9 purification by electro-elution

Size-fractionated rSmVAL9 was further purified by electro-elution (Model 422 Electro-Eluter; Bio-Rad) for 3 h at 60 mA in TGS buffer (25 mM Tris–HCl, 192 mM glycine, 0.1% SDS, pH 8.3). Afterwards, both upper and lower tank buffers were replaced with TG (25 mM Tris, 192 mM glycine, pH 8.3) and elution continued for a further 90 min to remove the SDS. Eluted samples were pooled and checked for purity by SDS–PAGE. The identity of rSmVAL9 was confirmed by in-gel trypsin digest (de Morree et al., 2010), followed by mass spectrophotometric (MS) analysis of extracted peptides by MALDI-TOF-MS and sequence identification by Mascot database queries as described (Meevissen et al., 2011). Protein concentration was determined by the Bradford method (Sigma–Aldrich) and aliquots of electro-eluted rSmVAL9 stored at −80 °C.

### 2.6 Murine rSmVAL9 antisera production

C57BL/6 mice were injected i.p. with 15 μg of electro-eluted rSmVAL9 (solubilised in 120 μl of TG buffer) emulsified in 80 μl of IMJECT Alum adjuvant (Thermo Scientific). A second immunisation was carried out at day 15 and a third and final booster delivered at day 36. Animals were sacrificed 4 days after the final immunisation and polyclonal, anti-rSmVAL9 antisera were obtained by cardiac puncture.

### 2.7 Affinity chromatography of SEA/glycopeptides

*Schistosoma mansoni* SEAs, protein-G Sepharose-linked 114-4D12-A monoclonal antibody (mAb) and the 114-4D12-A-binding SEA fraction were prepared as previously described (Robijn et al., 2007). Cysteine residues in the affinity-purified SEA glycoproteins (240 μg) were reduced and alkylated by mixing with 0.05 volumes of 200 mM dithiothreitol and incubating for 30 min at 56 °C. Thereafter, 0.2 volumes of 200 mM iodoacetamide were added followed by incubation for 30 min in the dark at 22 °C. Subsequently, the SEA fraction was incubated in 2 ml of 50 mM ammonium bicarbonate with 4 μg of trypsin (Promega, NL) at 37 °C overnight. In a second round of mAb 114–4D12-A affinity chromatography, the glycan epitope bearing glycopeptides within the trypsin digest were selected and subsequently purified by Zip-Tip_C18_ (Millipore, NL). The glycopeptides were eluted with 50% acetonitrile, 0.1% trifluoroacetic acid, dried in a stream of nitrogen and stored at -20 °C prior to further analysis by MS.

### 2.8 MS

For reversed-phase (RP) nano-scale liquid chromatography (LC) ion-trap-MS/MS analysis, the glycopeptide sample was applied to a reverse-phase column (PepMap, 3 μm, 75 μm × 100 mm; Dionex/LC Packings, NL) using an Ultimate nano-HPLC system, a Famos autosampler, and a Switchos trap-column system (Dionex/LC Packings). The column was equilibrated at room temperature with eluent A (0.1% formic acid in water) at a flow rate of 200 nl/min. After injection of the sample, elution conditions were switched to 10% solvent B (95% acetonitrile, 0.1% formic acid), followed by a gradient to 60% solvent B in 45 min and a subsequent isocratic elution of 10 min. The eluent was monitored by absorption at 215 nm.

The HPLC column was coupled to an Esquire HCT electrospray ionisation (ESI)-ion trap-MS (Bruker Daltonics, NL) containing an electron-transfer dissociation (ETD) module (PTM Discovery System™, NL). The MS instrument was operated in the positive-ion mode with an on-line nanospray source. For electrospray (1100–1250 V), capillaries (360 μm outer diameter (OD), 20 μm inner diameter (ID) with a 10 μm opening) from New Objective (Cambridge, MA, US) were used. The solvent was evaporated at 175 °C employing a nitrogen stream of 10 L/min. Ions from *m*/*z* 300 to *m*/*z* 1600 were registered in the MS mode. When operated in the auto MS/MS mode, registering ions from *m/z* 140 to 1800, each MS scan was followed by the acquisition of MS/MS spectra of up to three of the most abundant ions in the MS spectrum. For the ETD MS/MS experiment, the selected glycopeptide ion was isolated in the ion trap. Fluoranthene radical anions were formed by negative chemical ionisation (nCI) with methane as mediator. For the accumulation (typical accumulation time 5 ms) of fluoranthene reactant anions in the ion trap, the polarity was switched to negative mode. Glycopeptide cations and fluoranthene anions were incubated in the ion trap for 70 ms, allowing electron transfer, followed by the registration of the ETD fragment ion spectrum for *m/z* 140 to 3000. Selected MS/MS spectra were interpreted manually using Bruker Daltonics Data Analysis software (Bruker Daltonics).

### 2.9 SDS–PAGE and western blotting

SEA and rSmVAL9 were resolved by SDS–PAGE and transferred onto polyvinyl difluoride (PVDF, Millipore, UK) membranes as previously described (Hoffmann and Strand, 1996). Solubilised miracidia proteins, sporocyst proteins, LTP and EP were electrophoresed and electroblotted onto 0.2 μm nitrocellulose membranes as previously described (Wu et al., 2009). SEA or rSmVAL9 containing membranes were blocked in 50 mM Tris–HCl (pH 7.5)/0.3% Tween-20/5% skimmed milk powder before incubation with primary antibody (anti-rSmVAL9, normal mouse serum (NMS) or chronic mouse serum (CMS); dilutions indicated in figures) in 50 mM Tris–HCl (pH 7.5)/0.05% Tween-20/5% skimmed milk powder/150 mM NaCl for 3 h. Blots were washed three times in 50 mM Tris–HCl (pH 7.5) before incubating with secondary horseradish peroxidase-conjugated anti-mouse IgG antibody (Sigma), diluted 1:5000 for 1 h. After washing three times in 50 mM Tris–HCl, pH 7.5, blots were developed using ECL-Plus reagent (GE Healthcare, UK).

Miracidium, sporocyst, LTP and EP containing membranes were incubated in blocking buffer (TBST; 0.02 M Tris–HCl, 0.15 M NaCl, pH 7.5, 0.05% Tween-20, 5% BSA) overnight at 4 °C followed by exposure to 1:1000 dilution of mouse anti-rSmVAL9 in blocking buffer overnight at 4 °C. Membranes were then rinsed three times with TBS/0.05% Tween-20, incubated for 1 h in a 1:5000 dilution of alkaline phosphatase-conjugated goat anti-mouse IgG (Promega Corp., US), rinsed three times in TBS and developed using a NBT/BCIP AP chromogenic reagent (Thermo Scientific, USA). Quantitative comparisons of the steady state expression of SmVAL9 protein in miracidia and cultured sporocysts was accomplished by processing equal numbers of miracidia and sporocysts at different times post-cultivation for western blot analyses. Immunoreactive anti-SmVAL9 bands were scanned using a UV/colour densitometer (UVP Bioimaging Systems, USA) employing LabWorks 4.6 software. SmVAL9 band intensities were normalised to anti-actin (Developmental Studies Hybridoma Bank; http://dshb.biology.uiowa.edu/) densitometric values in each sample. The normalised means for three independent experimental replicates were statistically analysed by ANOVA and Tukey’s multiple means comparisons using GraphPad Prism (version 5).

### 2.10 SmVAL9 immunofluorescence analysis using scanning laser confocal microscopy (SLCM)

Fresh miracidia and cultured sporocysts were rinsed five times in CBSS+ on ice and fixed for 24 h in 1% PFA/1% Triton X-100 (Sigma–Aldrich) in sPBS at 4 °C. After fixation, larvae were washed five times in sPBS and once in blocking buffer (5% BSA/0.02% sodium azide), incubated in blocking buffer for 24 h at 4 °C and exposed to anti-rSmVAL9 (1:500 dilution in sPBS) for 24 h at 4 °C. Following primary antibody treatment, parasites were washed five times in sPBS and once in blocking buffer, and incubated in a secondary antibody mixture containing Alexa-fluor^®^488-conjugated goat anti-mouse IgG (4 mg/ml), Hoechst 33258 dye (50 mg/ml) and Alexa-fluor^®^546-conjugated phalloidin (7.5 mg/ml, Invitrogen) for 1 h at 22 °C in the dark. After five washes in sPBS, parasites were mounted on glass coverslips in Vectashield^®^ mounting medium (H-100, Vector Laboratories, Inc.) and imaged using an A1R laser scanning confocal microscope (Nikon Instruments Inc.) capable of scanning wavelengths of 408 nm, 488 nm and 561 nm for the excitation of Hoechst dye, Alexa Fluor^®^488 and Alexa Fluor^®^546, respectively. Negative controls included identically processed larvae in which: (i) NMS (1:500 diluted; Sigma–Aldrich) was substituted for the anti-SmVAL9 antibody and (ii) only the secondary Alexa-fluor^®^488-conjugated goat anti-mouse IgG antibody was used.

### 2.11 Bge cell/rSmVAL9 co-culture

Equal numbers of Bge cells were seeded into wells of 24-well culture plates and treated with rSmVAL9 (0.8 μg/ml in CBSS), CBSS alone or rSmVAL9 buffer (TG) alone in CBSS. After Bge cell cultures were exposed to recombinant protein or buffers for 24 h at 26 °C, cells were extracted with TRIzol^®^ reagent (Invitrogen, US) to isolate total RNA, followed by treatment with TURBO DNA-free (Ambion, US) to eliminate contaminating DNA. DNA-free, total RNA was then converted to cDNA using the SuperScript II first strand synthesis system kit (Invitrogen) as outlined by the manufacturer.

### 2.12 *Biomphalaria glabrata* matrix metalloproteinase 1 identification

A partial sequence of *B. glabrata* matrix metalloproteinase 1 (BgMMP1) was identified by tBLASTn searches of the dataset ‘bg_4_3_supercontigs’ from the *B. glabrata* genome assembly 4.3 (http://biology.unm.edu/biomphalaria-genome/index.html) using *M. musculus* MMP9 (NCBI accession No. NP_038627.1) and *Haliotis diversicolor* MMP1 (NCBI accession No. ABY87417.1) as query sequences. BgMMP1 was present on Contig2072, between 36,589 and 36,897 bp. Searches were performed on 09-01-13. NCBI conserved domain database searches (Marchler-Bauer et al., 2011) and multiple sequence alignments (MUSCLE (Edgar, 2004)) were performed using BgMMP1 to assess the conserved motifs/residues characteristic of matrix metalloproteinase protein family members. Amplification (forward 5′-ATG ACA CCC TCC TGT TTC TTC AAT CTA-3′; reverse 5′-TAA CTC GGT ACA CGA CCC GTA TTC CTT-3′) of a 307 bp BgMMP1 fragment (NCBI accession number KF287725) from *B. glabrata* embryonic (Bge) cell cDNA confirmed the transcription of this gene product. Molecular features of the cloned BgMMP1 fragment are summarised in Supplementary Fig. S1 and Supplementary Table S1.

### 2.13 ELISA

Sera from experimentally infected mice were assessed for total IgG response to rSmVAL9 by ELISA. Briefly, Immulon 4HBX (Nunc, UK) 96-well plates were coated over night at 4 °C with 100 ng/well of rSmVAL9 diluted in PBS. Plates were blocked with 200 μl of 5% milk powder/PBS/0.05% Tween-20 for 2 h at 37 °C. Blocking solution was aspirated and plates washed five times with PBS/0.05% Tween-20. Sera from individual mice were diluted 1:50 in PBS/0.05% Tween-20/1% BSA and 50 μl added to appropriate wells at 37 °C for 90 min. The plates were washed five times and then 50 μl of horse radish peroxidase (HRP) conjugated secondary antibody (Sigma) diluted 1:2000 in PBS/0.05% Tween-20/1% BSA were added to each well for 2 h at 37 °C. Plates were again washed five times and peroxidase signal developed for 14 min with 100 μl of 2,2’-azino-bis-3-ethylbenzothiazoline-6-sulphonic acid (ABTS) reagent (Kirkegaard and Perry Laboratories, KPL, USA). The reaction was terminated with 100 μl of 1% SDS and absorbance at 405 nm read immediately using a Polarstar Omega plate-reader (BMG Labtech, UK).

### 2.14 *Mus musculus* BMDMϕ/rSmVAL9 co-culture

Bone marrow was collected from the femurs of C57BL/6 mice and macrophage cultures prepared as previously described (Truscott et al., 2013). Cultures were allowed to equilibrate for 16 h then treated with rSmVAL9 (10 μg/ml) +/− polymixin B (10 μg/ml). Unstimulated cells and Tris–glycine (TG) buffer-only controls (the buffer used to electroelute rSmVAL9) were also maintained. After 20 h culture at 37 °C, media was removed and cells immediately lysed in 500 μl/well of TRIzol reagent (Invitrogen) for RNA extraction and cDNA synthesis as previously described (Truscott et al., 2013).

### 2.15 Semi-quantitative (sq) reverse transcription (sqRT)-PCR and quantitative (q)RT-PCR

rSmVAL9-induced BgMMP1 expression was assessed relative to the housekeeping Bgα actinin in Bge cells. sqRT-PCR primers were designed for the endogenous control Bgα-actinin (NCBI accession number AF484962) (forward 5′-ATG ACA CCC TCC TGT TTC TTC AAT CTA-3′; reverse 5′-TAA CTC GGT ACA CGA CCC GTA TTC CTT-3′) and BgMMP1 (NCBI accession number KF287725) (forward 5′-ATG ACA CCC TCC TGT TTC TTC AAT CTA-3′; reverse 5′-TAA CTC GGT ACA CGA CCC GTA TTC CTT-3′). Thirty-five cycles of PCR were used to determine BgMMP1 expression differences between Bge cell treatments (CBSS medium alone, TG buffer alone and rSmVAL9 in TG buffer) and the experiment was performed twice. BgMMP1 expression was normalised to Bgα-actinin expression by ImageJ densitometric analysis of stained amplicons.

qRT-PCR primers were designed for *M. musculus* MMP2, MMP9, MMP12, MMP13, MMP14 (MT-MMP1), MMP28, TIMP1 and TIMP2 as well as the endogenous control transcript *M. musculus* Hprt1 (Supplementary Table S2). qRT-PCR of *M. musculus* BMDMϕ cDNA was performed using SensiFAST SYBRGreen reagents (Bioline, UK) on a StepOnePlus Real-Time thermal cycler (Applied Biosystems, UK) using the fast protocol (10 μl reactions; 0.4 μM each primer, 2 μl of cDNA). Relative MMP and TIMP gene expression regulated by rSmVAL9 was calculated using the Pfaffl method (Pfaffl, 2001) where expression of each target gene in a test sample (*n *= 3) was calculated as a fold-change with respect to a calibrator cDNA population (untreated BMDMϕ) and normalised to expression of the reference gene Hprt1. Data was then transformed (−1) such that a fold-change of 0 demonstrates no change in gene expression between the compared cDNA populations.

## 3 Results

### 3.1 SmVAL9 is developmentally transcribed and post-translationally glycosylated

**Fig. 1:**
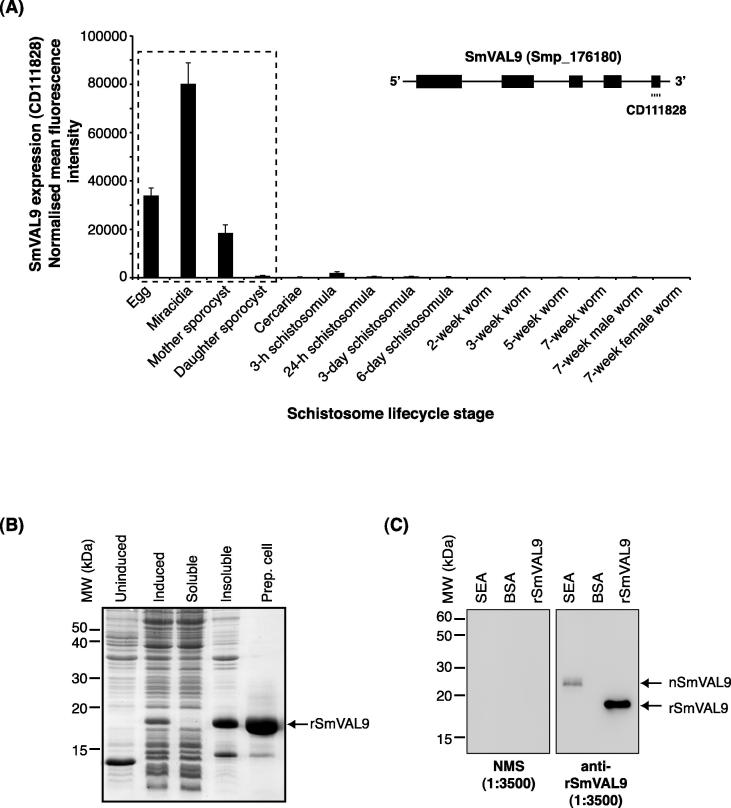
*Schistosoma mansoni* venom allergen-like 9 (SmVAL9) undergoes transcriptional regulation throughout schistosome development, is maximally expressed in miracidia and is post-translationally modified in eggs. (A) DNA microarray analysis of SmVAL9 expression throughout 15 lifecycle stages. Histogram represents normalised mean fluorescent intensities + S.D. (*n *= 3 replicates/lifecycle stage except adult female where *n *= 2) of SmVAL9 transcript abundance derived from oligonucleotide CD111828 as described previously (Fitzpatrick et al., 2009). Dashed box encloses schistosome lifecycle stages where SmVAL9 is maximally expressed. Inset drawing represents SmVAL9 (Smp_176180) gene organisation (five exons – black boxes; four introns – black lines) and localisation of oligonucleotide CD111828 to exon 5 (SchistoGeneDB v5.1). (B) Expression and purification of recombinant SmVAL9 (rSmVAL9) in *Escherichia coli* cells. The majority of rSmVAL9 is found in insoluble inclusion bodies after induction, allowing preparatory cell electrophoresis (Prep. Cell) to concentrate and purify large amounts of the protein for murine immunisations and other downstream studies. (C) Anti-rSmVAL9 recognises the 19.3 kDa rSmVAL9 immunogen and a ∼25 kDa post-translationally modified, native SmVAL9 (nSmVAL9) from soluble egg antigen. Pre-bleed normal mouse serum does not recognise either protein or the BSA control.

In a DNA microarray study designed to characterise the pattern of *S. mansoni* transcription throughout 15 lifecycle stages, we uncovered clusters of genes that were differentially expressed in snail-residing compared with mouse-residing parasitic forms (Fitzpatrick et al., 2009). In addition to SmVAL3 (Smp_193710) and SmVAL7 (Smp_199890), SmVAL9 (Smp_176180) was found within these gene lists. Closer interrogation of SmVAL9 transcription throughout schistosome maturation revealed peak expression in miracidia, followed by egg and mother sporocyst (48 h post in vitro transformation) developmental stages (Fig. 1A). These findings support the qRT-PCR determination of SmVAL9 abundance previously reported by Chalmers et al. (2008) and indicate that this group 1 SmVAL may be involved in similar activities within both snail and mammalian hosts. In order to test this hypothesis, a rSmVAL9 was expressed in *E. coli* cells and purified from insoluble inclusion bodies by preparatory cell electrophoresis (Fig. 1B). Polyclonal anti-rSmVAL9 antisera were subsequently raised in mice and initially used to probe an extract of SEAs by western blot analysis (Fig. 1C). While anti-rSmVAL9 recognised the 19.3 kDa rSmVAL9 immunogen, a shift in immunoreactivity to a 25 kDa protein was observed for native SmVAL9 (nSmVAL9) in the SEA sample (pre-bleed NMS not recognising either protein or the BSA control). This increase in molecular mass indicated that egg-derived nSmVAL9 is post-translationally modified and, as evidenced by the smear associated with the immunoreactive band, likely to be glycosylated.

**Fig. 2:**
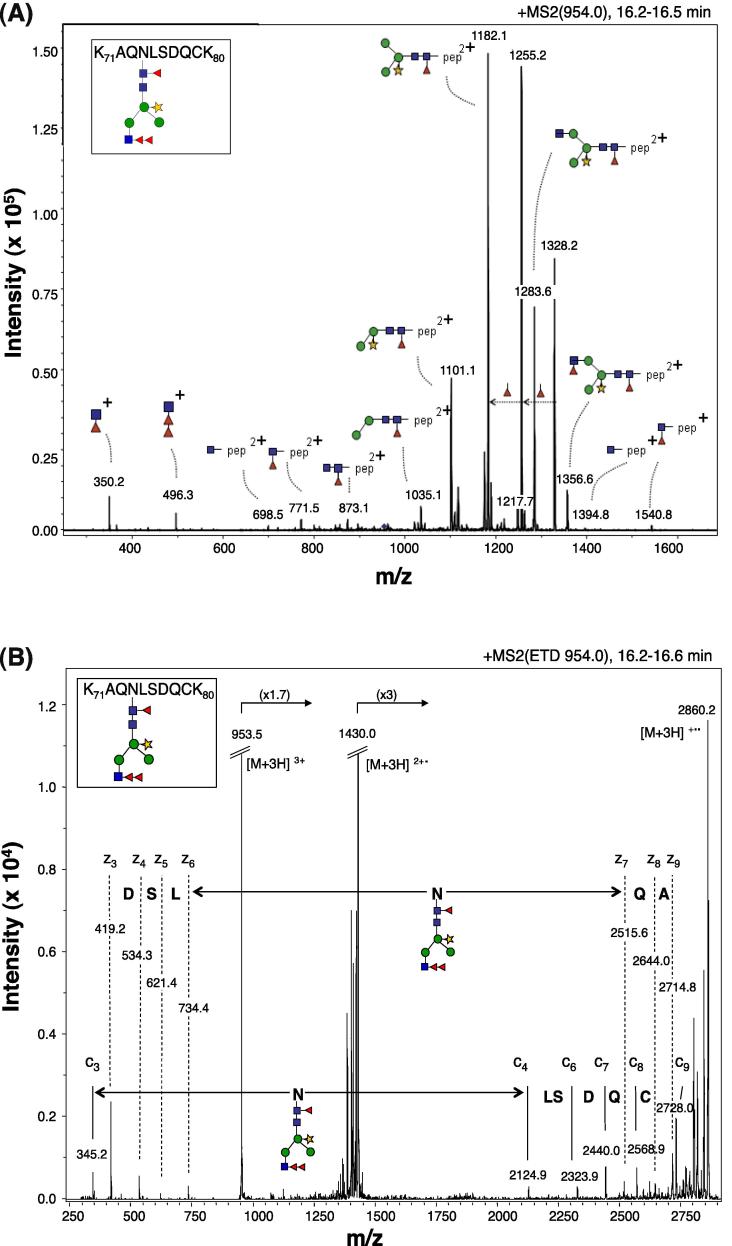
*Schistosoma mansoni* venom allergen-like 9 (SmVAL9) is an N-glycosylated soluble egg antigen containing a schistosome-specific, difucosyl modification. (A) Electrospray ionisation-ion trap MS/MS with collision-induced dissociation and with electron transfer dissociation (B) of the tryptic glycopeptide K_71_AQNLSDQCK_80_ from SmVAL9. The [M+3H]^3+^ parent ion at *m/z* 954.0 of the glycopeptide carrying a glycan of composition F_3_H_3_N_3_X_1_ was selected. Fragment ions are indicated in the figure. Monoisotopic masses are given. Square, *N*-acetylglucosamine; circle, mannose; triangle, fucose; star, xylose; pep, peptide moiety.

Native SmVAL9 glycosylation was verified by re-analysing data previously collected as part of a separate study aiming to identify and structurally characterise SEA glycoproteins that carry the unique schistosomal Fucα1–2Fucα1–3HexNAc (Fuc, fucose; HexNAc, *N*-acetylhexosaminine) glycan epitope recognised by the diagnostic antibody 114–4D12-A (Robijn et al., 2007). As part of this study, we isolated all tryptic glycopeptides in SEA that contained the Fucα1–2Fucα1–3HexNAc element by affinity chromatography (using antibody 114-4D12-A). The glycopeptides were applied to a RP-nano-LC column coupled to an ion-trap MS/MS system and fragmented in the auto-MS/MS mode. A parent-ion of *m/z* 954.0 [M+3H]^3+^ in the 16.2–16.5 min elution range was detected, giving rise to oxonium glycan fragment ions at *m/z* 350.2 [M+H]^+^ (deoxyHex_1_HexNAc_1_, F_1_N_1_) and *m/z* 496.3 [M+H]^+^ (F_2_N_1_), which indicate the presence of the terminal difucosylated HexNAc element. Further inspection of the collision-induced dissociation (CID) MS/MS spectrum of the parent ion *m/z* 954.0 [M+3H]^3+^, which is dominated by glycosidic linkage cleavages (Wuhrer et al., 2007), indicates the presence of a glycan with the composition F_3_H_3_N_3_X_1_ (F, fucose; H, hexose; N, HexNAc; X, xylose) linked to a peptide of 1192 Da (Fig. 2A). [M+2H]^2+^ fragment ions are observed for this peptide carrying a single HexNAc (*m/z* 698.5) or a Fuc_1_HexNAc_1_ disaccharide (*m/z* 771.5) derived from a mono-fucosylated chitobiose core structure of an Asn-linked glycan. Furthermore, a dominant series of [M+2H]^2+^ fragment ions at *m/z* 1356.6 (F_2_H_3_N_3_X_1_-peptide), 1238.6 (F_1_H_3_N_3_X_1_-peptide), 1182.1 (F_1_H_3_N_2_X_1_-peptide), 1101.1 (F_1_H_2_N_2_X_1_-peptide) and 1035.1 (F_1_H_2_N_2_-peptide) are fully in line with the presence of an N-glycan with a fucosylated, xylosylated trimannosyl core, carrying one difucosylated N-acetylglucosamine residue forming the mAb 114-4D12-A epitope. The [M+2H]^2+^ signals observed at *m/z* 1255.2 (F_1_H_3_N_2_X_1_-peptide) and 1328.2 (F_1_H_3_N_2_X_1_-peptide) arise from the fucose rearrangements commonly observed in CID-MS/MS of proton adducts of glycans and glycoconjugates (Wuhrer et al., 2006b).’

To obtain information on the glycopeptide sequence recognised by mAb 114-4D12-A, an ETD MS/MS spectrum of the same parent ion *m/z* 954.0 [M+3H]^3+^ was recorded (Fig. 2B) in which peptide cleavages are predominantly observed while leaving the glycosidic linkages intact (Wuhrer et al., 2007). The c′-type as well as z^•^-type ions arising from peptide bond cleavages provide partial sequences of the peptide backbone from the N- and C-terminal side, respectively. As annotated in Fig. 2B, the LSDQC sequence can be read directly from the ion c_4_-c_9_ ion series, while the signals indicated with z_3_-z_9_ read the DSLN(glycan)QA sequence, including the mass increment of 1665 Da accountable to the glycan. Submitting sequence tag AQNLSDQC to the NCBI BLAST (nr) database returned a 100% coverage and identity hit with SmVAL9 (XP_002582201). This sequence is part of the SmVAL9 tryptic peptide (one missed cleavage) K_71_AQNLSDQCK_80_, containing the N-glycosylation consensus sequence NLS. Taken together, the MS/MS data indicated that SEA-derived SmVAL9 is modified at Asn_74_ with a core xylosylated, core fucosylated N-glycan carrying one terminal difucosyl *N*-acetylglucosamine residue that forms the epitope recognised by mAb 114-4D12-A.

### 3.2 E/S SmVAL9 up-regulates *B. glabrata* MMP1 expression

**Fig. 3:**
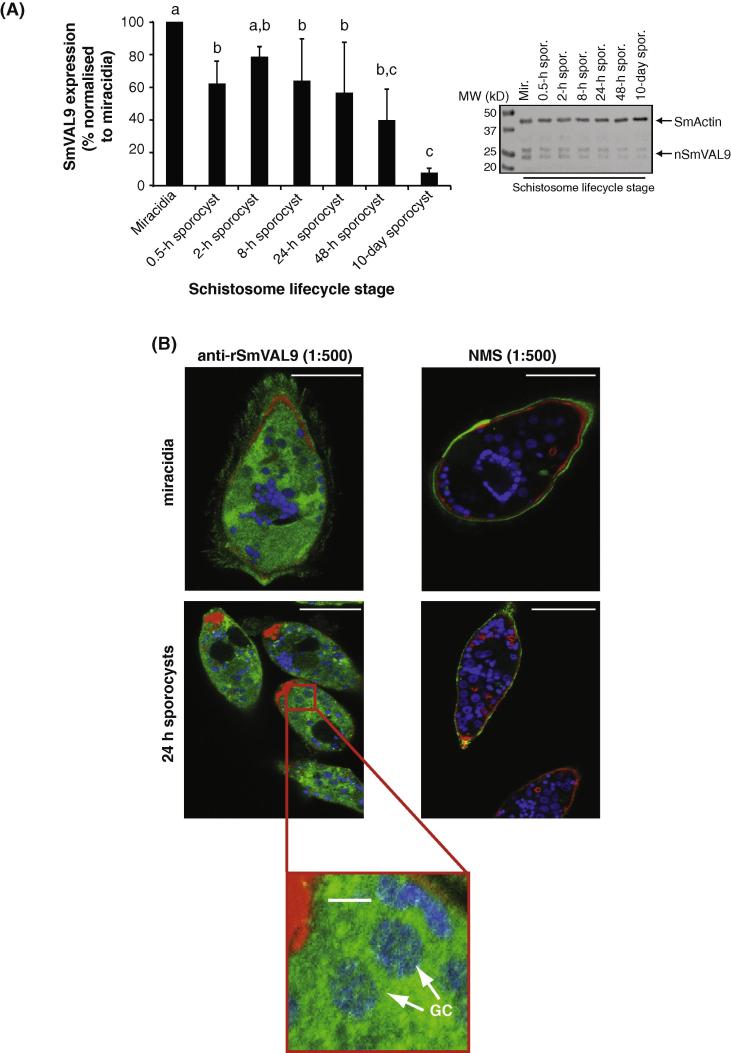
*Schistosoma mansoni* venom allergen-like 9 (SmVAL9) is translationally regulated in intra-molluscan *S. mansoni* lifecycle stages and is found throughout the parasite’s parenchyma, perikarya and putative germinal cells. (A) Quantitative assessment of SmVAL9 protein expression in free-swimming miracidia and primary sporocysts at different times post in vitro cultivation (0.5 h to 10 days). Histogram on the left represents the relative means + S.D. of steady-state sporocyst SmVAL9 protein normalised to miracidia, as a function of time in culture. Means were generated by densitometric analyses of western blots displaying immunoreactive native (n)SmVAL9 in miracidia and sporocysts (*n *= 3; representative figure is shown). All larval values were normalised to anti-SmActin reactivities (loading control) prior to statistical analysis (ANOVA; *F*_6,2_ = 8.59; *P *= 0.0048). Mean values sharing the same letter are not significantly different as determined by Tukey’s multiple comparison test. (B) Immunocytochemical localisation of SmVAL9 in *S. mansoni* miracidia and 24 h cultured primary sporocysts by laser scanning confocal microscopy. Specific immunoreactivity was observed diffusely distributed in the parenchyma, perikarya and putative germinal cells (insert, 24 h sporocyst) of miracidia and sporocysts. Surface immunostaining in normal mouse serum and secondary antibody only controls (data not shown) likely represents non-specific bound mouse IgG acquired in ovo. Bars = 50 μm; insert bar = 10 μm. GC, germinal cells.

To verify whether SmVAL9 translation follows the transcriptional pattern observed during schistosome intramolluscan development (Fig. 1A), we performed a quantitative western blot analysis using anti-rSmVAL9 and protein extracts derived from miracidia as well as in vitro cultured sporocysts harvested at different times (0.5 h–10 days) post-transformation (Fig. 3A). Here, peak SmVAL9 protein abundance was found in miracidia with diminishing amounts of this group 1 VAL being found in sporocyst stages during development. These western blot results confirmed the DNA microarray studies depicted in Fig. 1A and demonstrated that SmVAL9 transcription and translation patterns correlate. Interestingly, anti-rSmVAL9 analysis of miracidia and sporocyst lifecycle stages indicated two distinct immunoreactive proteins (inset western blot, Fig. 3A). As SmVAL9 bears sequence similarity to other SmVALs (SmVAL5, 15, 26, 27, 28 and 29) known to be present in miracidia/sporocyst developmental forms (Wu et al., 2009), it is possible that one of these bands may represent a cross-reactive family member. The most likely candidate to be detected by the anti-rSmVAL9 antisera is SmVAL29, which shares 57% sequence identity and 71% sequence similarity. Further studies would be necessary to confirm this hypothesis, however, we cannot rule out this possibility.

We next utilised the anti-rSmVAL9 antisera to define SmVAL9 localisation within miracidia and 24 h cultured sporocysts (Fig. 3B). Here, clear anti-rSmVAL9 immunoreactivity (green) was observed within both miracidium as well as sporocyst samples and was associated with a diffusely distributed pattern throughout the parenchyma, perikarya and putative germinal cells (inset box in Fig. 3B of the 24 h sporocysts). Additional anti-rSmVAL9 immunoreactivity was also associated with the miracidia cilia. Pre-bleed NMS reacted with the surface of both miracidia and sporocyst samples and is likely due to non-specifically bound mouse IgG acquired in ovo.

**Fig. 4:**
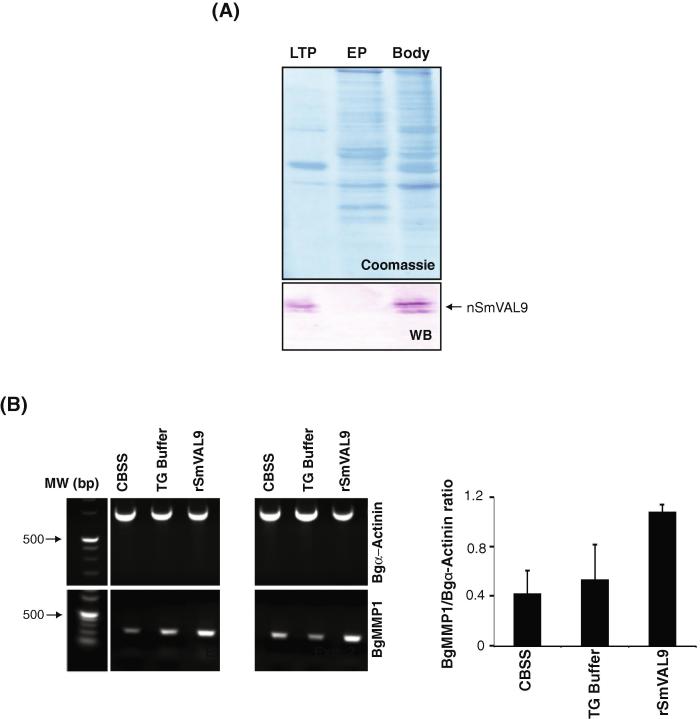
*Schistosoma mansoni* venom allergen-like 9 (SmVAL9) released during the process of miracidia to sporocyst transformation induces *Biomphalaria glabrata* matrix metalloproteinase expression. (A) Native SmVAL9 is released into medium during early larval cultivation. Western blot showing immunoreactive native (n)SmVAL9 in culture supernatant containing larval transformation proteins and in whole body homogenates (Body), but not in shed epidermal plates. Absence of epidermal plates immunoreactivity is not due to differences in sample protein loads as indicated by intensities of Coomassie blue staining. (B) In vitro exposure of the *B. glabrata* embryonic (Bge) cell line to rSmVAL9 (0.8 μg/ml) stimulates matrix metalloproteinase 1 (BgMMP1) transcription. Using *B. glabrata* α (Bgα)-actinin as a reference, relative intensity of the BgMMP1 amplicon was greater in rSmVAL9-exposed Bge cells compared with cells treated with snail saline alone (CBSS) or rSmVAL9 Tris–glycine buffer alone (TG buffer). Results of two independent experiments are shown with inset histogram representing mean densitometric values of BgMMP1/Bgα-actinin expression + S.D.

Due to the presence of anti-rSmVAL9 reactivity to miracidia cilia (Fig. 3B) and the previously described proteomic identification of SmVAL9 peptides in miracidia/sporocyst transformation products (Wu et al., 2009), we next wanted to assess what components of our miracidia/sporocyst transformation products contained native SmVAL9 (Fig. 4). Here, larval transformation proteins (LTP), shed epidermal plates (EP) and remaining parasite bodies (Body) were obtained from a typical in vitro miracidia transformation procedure and incubated with anti-rSmVAL9 antisera during western blot analysis of solubilised proteins (Fig. 4A). From these results, two anti-rSmVAL9 immunoreactive proteins were found in the LTP and Body samples, but not in the EP preparation confirming that SmVAL9 was indeed released into the immediate environment during larval transformation. These results also indicated that most, if not all, SmVAL9 was found within non-epidermal plate containing material, but E/S SmVAL9 could adhere to cilia in intact miracidia (refer to Fig. 3B).

Having established that SmVAL9 was excreted/secreted during the process of miracidium to sporocyst transformation (Figs. 3 and 4A) and a previous study demonstrating that mammalian RTVP-1 (a SmVAL9 homolog) up-regulated gelatinase A/MMP2 expression in glioma cells and influenced the invasion of astocytic tumors (Rosenzweig et al., 2006), we postulated that a conserved function in tissue remodelling was possible for E/S SmVAL9. Here, using the Bge cell line, we specifically examined the ability of rSmVAL9 to influence the transcription of a newly discovered *B. glabrata* matrix metalloproteinase (BgMMP1, NCBI Accession number KF287725, Supplementary Fig. S1 and Supplementary Table S1). Identified from the publically available *B. glabrata* genome (http://biology.unm.edu/biomphalaria-genome/index.html), the partial BgMMP1 sequence encodes the conserved catalytic domain of MMPs including the HxxGHxxxxxH zinc-binding motif and metalloproteinase-specific serine (S) residue (Bode et al., 1993). After 24 h of Bge/rSmVAL9 co-culture, we consistently observed an up-regulation of BgMMP1 expression when compared with Bge cultures co-cultured with CBSS or TG buffers only (Fig. 4B, two representative experiments illustrated).

### 3.3 E/S SmVAL9 is immunogenic during murine schistosomiasis and differentially regulates *M. musculus* MMP and TIMP expression in BMDMϕ cultures

**Fig. 5:**
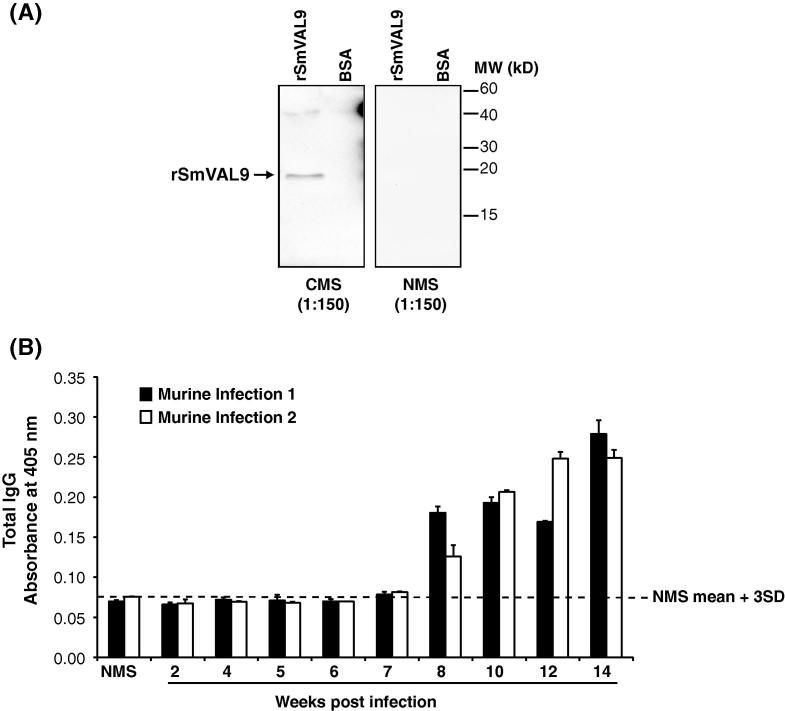
*Schistosoma mansoni* venom allergen-like 9 (SmVAL9) is an immunogenic IgG target recognised during experimental murine schistosomiasis. (A) Ten micrograms of recombinant (r)SmVAL9 or BSA were electrophoresed, blotted onto polyvinyl difluoride and probed with chronic mouse serum; obtained from mice experimentally infected for 14 weeks; 50 cercariae/mouse or normal mouse serum (both at 1:150). A 19.3 kDa, immunoreactive protein is specifically observed in the rSmVAL9 lane when probed with chronic mouse serum, but not normal mouse serum. (B) ELISA measurement of anti-rSmVAL9 IgG reactivity in mice experimentally infected (50 cercariae/mouse) with *S. mansoni*. Histograms represent mean (+S.D.) anti-rSmVAL9 total IgG absorbance (four to six animals/time-point) measured in mice at 2–14 weeks p.i. from two repeat experiments (black histograms, murine infection 1; white histograms, murine infection 2). Dashed line represents mean anti-rSmVAL9 reactivity + 3 S.D. derived from normal mouse serum.

As SmVAL9 is a glycoprotein found in SEA (Figs. 1 and 2) and is a component of E/S products released from eggs (Cass et al., 2007), we next investigated whether the definitive mammalian host was capable of recognising this group 1 VAL during experimental schistosomiasis (Fig. 5). Here, utilising pooled serum samples obtained from mice infected with *S. mansoni* for 14 weeks (CMS), we determined that rSmVAL9 was indeed an IgG target during chronic schistosomiasis (Fig. 5A). Pooled sera collected from uninfected mice (NMS) did not recognise rSmVAL9 in these same western blot experiments. A western blot control protein used in these experiments, BSA, also was not recognised by either CMS or NMS.

We subsequently extended these preliminary western blot studies to an ELISA format (wells coated with rSmVAL9), which would allow for a greater number of p.i. time-points to be measured for anti-rSmVAL9 reactivity. Here, sera samples (four to six mice/time-point) derived from mice experimentally infected with *S. mansoni* for at least 2 and up to 14 weeks were used (Fig. 5B). While schistosome egg production normally begins at week 5 p.i. in the murine host, anti-rSmVAL9 IgG responses lagged behind oviposition and were not measurable (above background; NMS mean + 3S.D.) until week 8 p.i. This was observed in two independent experiments (black histograms, murine infection 1; white histograms, murine infection 2). From week 8, a gradual rise in anti-rSmVAL9 IgG titres was observed in both replicate experiments until a peak anti-rSmVAL9 response was measured in the final 14-week p.i. time-point.

**Fig. 6:**
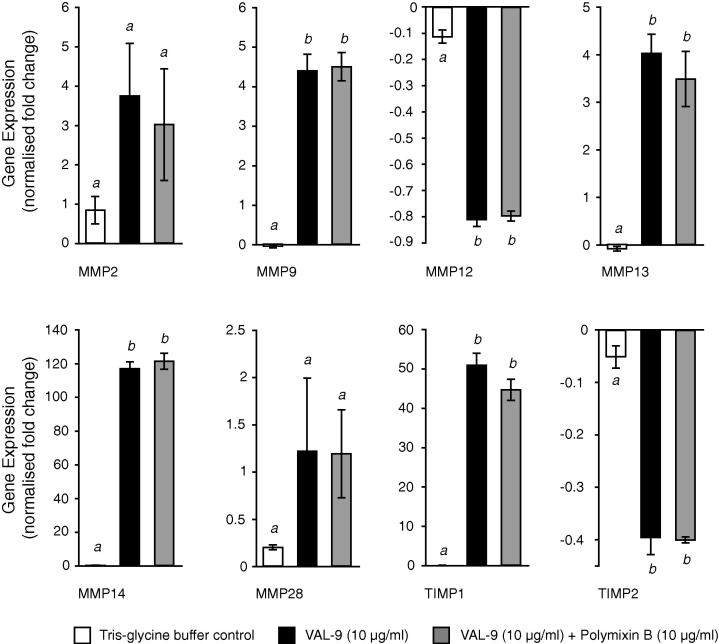
*Schistosoma mansoni* venom allergen-like 9 (SmVAL9) differentially activates matrix metalloproteinase and tissue inhibitors of metalloproteinase expression in murine bone marrow-derived macrophages. Bone marrow-derived macrophage cultures were stimulated with Tris–glycine buffer (recombinant (r)SmVAL9 solvent; white bars), rSmVAL9 (10 μg/ml; black bars) or rSmVAL9 (10 μg/ml) in the presence of polymyxin B (10 μg/ml; grey bars) for 20 h. The relative expression of MMP2, MMP9, MMP12, MMP13, MMP14 (MT-MMP1), MMP28, TIMP1 and TIMP2 was measured via quantitative reverse transcription-PCR and compared with untreated macrophage populations. Histograms represent mean MMP/TIMP expression (normalised fold change) ± S.E.M. (*n *= 3). Statistical significance was determined using ANOVA tests (*P *< 0.01) and post hoc *t*-tests where appropriate. Mean values sharing the same italicised letter are not significantly different.

Having determined that SmVAL9 is recognised during murine schistosomiasis at time-points after egg deposition, we next asked whether (similar to Bge cells) this E/S group 1 VAL released in the definitive host was capable of influencing the expression of genes involved in tissue remodelling (Fig. 6). As a model to test this hypothesis, we chose BMDMϕ derived from C57BL/6 mouse femurs (Truscott et al., 2013) and co-cultured those in the presence of rSmVAL9 (+/− polymyxin B). We specifically assessed the capacity of rSmVAL9 to differentially regulate the expression of key matrix metalloproteinases (MmMMP2, MmMMP9, MmMMP12, MmMMP13, MmMMP14 and MmMMP28) as well as tissue inhibitors of metalloproteinases (MmTIMP1 and MmTIMP2) involved in extracellular matrix remodelling and hepatic fibrosis during schistosomiasis (Vaillant et al., 2001; Sandler et al., 2003; Madala et al., 2010; Anthony et al., 2013). Here, we found that rSmVAL9 stimulated the transcription of genes positively involved in degradation of the extracellular matrix (MMP9, MMP13, MMP14/MT-MMP1 and to a lesser degree MMP2) while simultaneously suppressing the transcription of genes negatively associated with the same process (TIMP2, MMP12) (Madala et al., 2010; Cardeal et al., 2012; Chellaiah and Ma, 2013) (Fig. 6). TIMP1 was also highly induced in rSmVAL9 stimulated BMDMϕ, while MMP28 transcription was minimally affected. Polymyxin B did not affect the rSmVAL9 transcriptional alterations in BMDMϕ suggesting that our rSmVAL9 produced in *E. coli* was free from contaminating endotoxins, the presence of which could have complicated the interpretation of MmMMP/MmTIMP expression in BMDMϕ cultures (Hanania et al., 2012). Taken together (and similar to what was found in the Bge system, Fig. 4), the rSmVAL9/BMDMϕ co-culture experiments suggested that E/S SmVAL9 manipulates host cell transcription of MMPs/TIMPs and this activity may contribute to an environment suitable for egg translocation and lifecycle transmission.

## 4 Discussion

Functional characterisation of the schistosome molecules/metabolites involved in molluscan and mammalian host interactions will help reveal how these highly successful parasites establish and maintain the biomedically-important, neglected tropical disease schistosomiasis. In this regard, recent studies of polymorphic mucins (Mone et al., 2010), E/S helminth defence molecules (Thivierge et al., 2013), omega-1 (Everts et al., 2009; Steinfelder et al., 2009), IPSE/alpha-1 (Schramm et al., 2003) and regurgitated haemozoin (Truscott et al., 2013) have all detailed the contributory role of developmentally-regulated schistosome gene products/metabolites in manipulating the (immuno) biology of intermediate or definitive host cells. Extending these functional investigations to other schistosome biomolecules found at the host/parasite interface will undoubtedly increase the number of targets considered for next-generation chemotherapeutic (drugs), immunotherapeutic (immunomodulators) or immunoprophylactic (vaccines) strategies. As part of our ongoing activities in this area (Fitzpatrick et al., 2009; Geyer et al., 2011; Everts et al., 2012; Meevissen et al., 2012; Yoshino et al., 2012; Peterson et al., 2013; Truscott et al., 2013), we herein describe the activity of a SmVAL9 molecule on both Bge cells and *M. musculus* BMDMϕ.

The 29 SmVALs encoded in the *S. mansoni* genome (v5.2) are related to an often described, but poorly understood, family of proteins (Sperm Coating Protein/Tpx-1/Ag5/PR-1/Sc7; SCP/TAPS) distributed throughout the Archaea, Eubacteria and Eukarya (Chalmers and Hoffmann, 2012). Within the parasitic Nematoda, some E/S SCP/TAPS members have been implicated in immunomodulatory activities including platelet aggregation inhibition (Del Valle et al., 2003), neutrophil chemotaxis manipulation (Bower et al., 2008), neutrophil binding (Moyle et al., 1994) and angiogenesis stimulation (Tawe et al., 2000). It is thought that these activities help the invading L3s establish infection, modulate potentially deleterious host immune responses and induce environmental conditions suitable for survival (Cantacessi et al., 2009). As schistosomes encounter similar immunological/environmental challenges during lifecycle progression through molluscan and mammalian hosts, we postulated that E/S group 1 SmVALs could function in a similar immunomodulatory/environmentally-altering vein. SmVAL9 was chosen as the representative group 1 protein with which to investigate this hypothesis due to its presence in both mammalian-host inhabiting egg (Cass et al., 2007) and molluscan-host residing miracidia/sporocyst excretions/secretions (Wu et al., 2009).

Our results confirm the presence of SmVAL9 in both egg and miracidia/sporocyst lifecycle stages (Figs. 1 and 2). However, several new observations about this developmentally-regulated group 1 VAL were recorded in this study. While many O-linked glycans found in SEA contain the double fucose containing epitope described here (Robijn et al., 2007) and multi-fucosylated elements are present on a subset of SEA-derived N-linked glycans (Khoo et al., 1997; Hokke et al., 2007), we have now identified for the first time a specific carrier, SmVAL9, that presents this particular Fucα1–2Fuc element (Fig. 2). Furthermore, full MS/MS characterisation of the SmVAL9 tryptic N-glycopeptide illustrates a glycosylation quite distinct to the glycosylation patterns described for three other major egg antigens including omega-1, IPSE/alpha-1 and kappa-5. E/S omega-1 and IPSE/alpha-1 both contain Galβ1–4(Fucα1–3)GlcNAc (Lewis X) antigens aaa(Wuhrer et al., 2006a; Meevissen et al., 2010), while non-E/S kappa-5 contains multiple GalβNAc1–4GlcNAc (LDN) termini (Meevissen et al., 2011). The absence of Lewis X and LDN elements, together with the presence of the difucosylated terminal N-linked motif, suggests that SmVAL9 interacts with one or more C-type lectin receptor different from those that bind omega-1, IPSE/alpha-1 or kappa-5 (Meevissen et al., 2012). A lectin receptor that preferentially binds the difucosyl element of SmVAL9 has not yet been identified.

When considering where anti-rSmVAL9 reactivity is found within miracidia/sporocyst lifecycle stages, our results indicate diffuse SmVAL9 localisation to parenchyma, perikaya and germinal cells (Fig. 3B). As the miR-137 target RTVP-1 (a SCP/TAPS protein related to SmVAL9) has recently been found to influence the self-renewal and differentiation of glioblastoma stem cells (Bier et al., 2013), SmVAL9 localisation to this parasite cell population is intriguing. This is especially true in light of the recent description of SmVAL22 expression in sporocyst germinal cell populations (Wang et al., 2013). Our data supports the association of SmVALs in sporocyst germinal cells and expands the repertoire found in this important self-renewing schistosome cell population maintained throughout parasite development (Collins et al., 2013). Importantly, SmVAL9 excretion/secretion during miracidia/sporocyst transformation (Fig. 3C) as well as SmVAL9 excretion/secretion from eggs (Cass et al., 2007) indicates another possible function(s) (additional to germinal cell biology) related to parasite/host interactions. We therefore explored this hypothesis using two different host cell models (molluscan Bge cells and mammalian BMDMϕ).

In both models (Figs. 4B and 6), the ability of rSmVAL9 to differentially regulate host cell MMP expression was specifically explored due to the capacity of RTVP-1 to induce the up-regulation and secretion of MMP2 from glioma cells (Rosenzweig et al., 2006). This RTVP-1 mediated MMP2 secretion facilitates ECM remodelling and influences the invasion of astrocytic tumors. As miracidia to sporocyst transformation in *B. glabrata* involves parasite invasion and host ECM remodelling (Borges and Andrade, 2003), a likely conserved role for E/S SmVAL9 in regulating MMP expression was postulated. After first identifying a bona fide *B. glabrata* MMP (BgMMP1) in the *B. glabrata* genome (http://biology.unm.edu/biomphalaria-genome/index.html), with high sequence similarity to membrane-type 3 MMP (also known as MMP16; (Shi et al., 2008; Sabeh et al., 2009)) database hits (Supplementary Fig. S1 and Supplementary Table S2), we assessed the capacity of rSmVAL9 to regulate its expression in Bge cells by sqRT-PCR (Fig. 3D). Clear induction of BgMMP1 transcription was seen in Bge cells stimulated with rSmVAL9 at a level greater than that measured in Bge cultures stimulated with buffer controls and this activity was independent of the presence of the double fucose containing glycan found on native SmVAL9 (Fig. 2). It is known that mammalian MMP16 activates MMP2 into an active, ECM degrading zymogen (Astarci et al., 2012), favouring the invasive potential of cells from one locality to another (Stamenkovic, 2003). While we have yet to elucidate the full MMP repertoire (including a MMP2 like homolog) encoded in the *B. glabrata* genome or to determine whether rSmVAL9-induced BgMMP1 (MMP16-like homolog) transcription correlates with enzymatic activity in Bge cells, our cautious interpretation of these findings implicates a potential function of E/S SmVAL9 in creating an environment suitable for miracidia invasion and sporocyst migration throughout *Biomphalaria* tissues. Another potential benefit for larval SmVAL9 induction of MMPs stems from the fact that this enzyme family contains conserved haeme-binding domains (haemopexin-like domains; (Piccard et al., 2007)) capable of binding/sequestering Fe-containing haem moieties comprising snail haemoglobin, thereby disrupting potentially lethal anti-parasite redox reactions (Bayne et al., 2001; Tolosano and Altruda, 2002).

Additional evidence to support a host modulatory role for SmVAL9 was derived from parallel experiments in rSmVAL9/BMDMϕ co-cultures where the expression of several MMPs and TIMPs were investigated by qRT-PCR (Fig. 6). A previous study demonstrated that SEA was capable of inducing MMP9 expression in LX-2 cells (a human hepatic stellate cell line) with this activity postulated to originate in the excretory/secretory products released from the eggs (Anthony et al., 2013). Furthermore, as tissue-degrading MMP9, MMP2 and MMP13 function is regulated by MMP12 activity during granulomatous pathology (Madala et al., 2010), E/S SmVAL9 from eggs could behave as a pro-ECM remodelling factor by manipulating the reciprocal expression of these MMPs. Our findings are entirely consistent with this hypothesis and those results reported by Madala et al. (2010), Cardeal et al. (2012) and Chellaiah and Ma (2013), suggesting that excretory/secretory SmVAL9 activates the differential expression of MMPs/TIMPs instrumental in tissue remodelling. This activity likely contributes to an environment suitable for the translocation of eggs across the intestinal lumen and/or the development of immunologically-regulated granulomatous pathology (Hoffmann et al., 2002). Host IgG recognition of SmVAL9 may participate in regulation of these processes (Fig. 4) by decreasing the amount of circulating/tissue-trapped SmVAL9. However, similar to the Bge studies, these interpretations need tempering until active MMP secretion from rSmVAL9 stimulated BMDMϕs is characterised. Irrespective, we contend that both Bge and BMDMϕ models demonstrate that SmVAL9 is capable of differentially regulating host cell MMP/TIMP transcription and that this activity may participate in the co-evolved survival strategies employed by schistosomes during host/parasite interactions.

## Appendix A Supplementary data

Supplementary Fig. S1 Characterisation of a *Biomphalaria glabrata* matrix metalloprotease 1 (BgMMP1) sequence. (A) Protein domain search results against BgMMP1 amino acid sequence using the NCBI conserved domain search. (B) Multiple sequence alignment of (BgMMP1) partial sequence with related matrix metalloproteases from *Caenorhabditis elegans* (CeZMP; [NP_741156]), *Drosophila melanogaster* (DmMMP1; [AAF47255.3] & DmMMP2; [AAS64885.1]), *Haliotis diversicolor* (HdMMP1; [ABY87417.1]), *Homo sapiens* (HsMMP2; [1QIB_A]), *Mus musculus* MmMMP16 [EDL05586.1] and *Felis catus* (FcMMP16; [AAR03975.1]). Numbers at the beginning of each motif represent amino acid positions and at each position the most conserved residues are further shaded in black, semi-conserved residues are highlighted grey and non-conserved amino acids are kept white. The functionally important HxxGHxxxxxH motif and serine (S) and methionine (M) residues (Bode et al., 1993), are indicated by an asterisk (∗) above the alignment.

Supplementary Table S1 tBLASTn search results using a partial protein sequence encoded by *Biomphalaria glabrata* matrix metalloproteinase 1 (BgMMP1) sequence against the NCBI nucleotide database.

Supplementary Table S2 Quantitative reverse transcription (qRT)-PCR primers used in this study.
