# Supplementary figures and images for: Excreted/secreted Schistosoma mansoni venom allergen-like 9 (SmVAL9) modulates host extracellular matrix remodelling gene expression
Source: Int J Parasitol. 2014 Jul;44(8):551–63. doi: 10.1016/j.ijpara.2014.04.002 (PMC4079936; doi:10.1016/j.ijpara.2014.04.002)

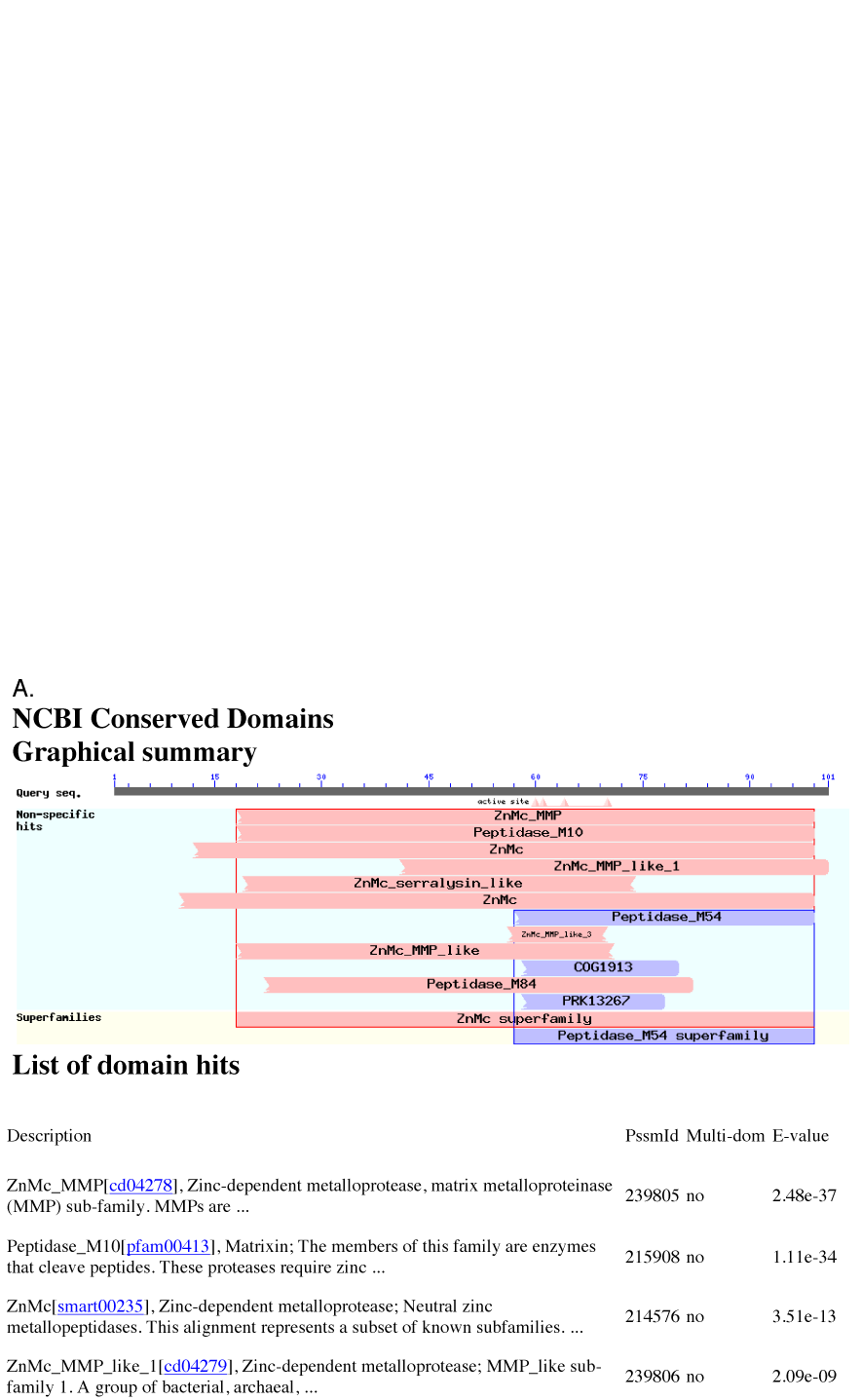


(A)

(B)


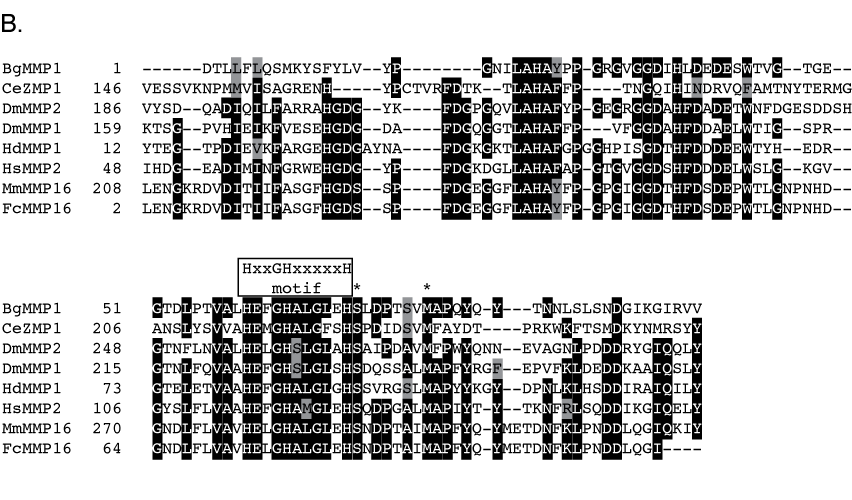

Supplement: Supplementary Fig. S1 — Characterisation of a Biomphalaria glabrata matrix metalloprotease 1 (BgMMP1) sequence. (A) Protein domain search results against BgMMP1 amino acid sequence using the NCBI conserved domain search. (B) Multiple sequence alignment of (BgMMP1) partial sequence with related matrix metalloproteases from Caenorhabditis elegans (CeZMP; [NP_741156]), Drosophila melanogaster (DmMMP1; [AAF47255.3] & DmMMP2; [AAS64885.1]), Haliotis diversicolor (HdMMP1; [ABY87417.1]), Homo sapiens (HsMMP2; [1QIB_A]), Mus musculus MmMMP16 [EDL05586.1] and Felis catus (FcMMP16; [AAR03975.1]). Numbers at the beginning of each motif represent amino acid positions and at each position the most conserved residues are further shaded in black, semi-conserved residues are highlighted grey and non-conserved amino acids are kept white. The functionally important HxxGHxxxxxH motif and serine (S) and methionine (M) residues (Bode et al., 1993), are indicated by an asterisk (∗) above the alignment. [file mmc1.doc]
