# Supplementary material for: Excreted/secreted Schistosoma mansoni venom allergen-like 9 (SmVAL9) modulates host extracellular matrix remodelling gene expression
Source: Int J Parasitol. 2014 Jul;44(8):551–63. doi: 10.1016/j.ijpara.2014.04.002 (PMC4079936; doi:10.1016/j.ijpara.2014.04.002)
Supplement: Supplementary Table S1 — tBLASTn search results using a partial protein sequence encoded by Biomphalaria glabrata matrix metalloproteinase 1 (BgMMP1) sequence against the NCBI nucleotide database. [file mmc2.docx]

| **Hit number** | **Description** | **Query cover** | **E value** | **Identity** | **Accession number** |
| --- | --- | --- | --- | --- | --- |
| 1 | PREDICTED: *Maylandia zebra* matrix metalloproteinase-20-like (LOC101486079), mRNA | 83% | 2e-24 | 55% | XM_004543921.1 |
| 2 | *Felis catus* membrane type matrix metalloproteinase 16 mRNA, partial cds | 79% | 9e-24 | 61% | AY250763.1 |
| 3 | PREDICTED: *Anolis carolinensis* matrix metallopeptidase 16 (membrane-inserted) (mmp16), mRNA | 82% | 1e-23 | 59% | XM_003219534.1 |
| 4 | PREDICTED: *Ficedula albicollis* matrix metallopeptidase 16 (membrane-inserted) (MMP16), mRNA | 82% | 2e-23 | 59% | XM_005042275.1 |
| 5 | PREDICTED: *Tursiops truncatus* matrix metallopeptidase 16 (membrane-inserted) (MMP16), mRNA | 82% | 2e-23 | 59% | XM_004314196.1 |
| 6 | PREDICTED: *Anas platyrhynchos* matrix metallopeptidase 16 (membrane-inserted) (MMP16), mRNA | 82% | 3e-23 | 59% | XM_005016732.1 |
| 7 | PREDICTED: *Taeniopygia guttata* matrix metallopeptidase 16 (membrane-inserted) (MMP16), mRNA | 82% | 3e-23 | 59% | XM_002199847.2 |
| 8 | *Gallus gallus* matrix metallopeptidase 16 (membrane-inserted) (MMP16), mRNA >gb\|U66463.1\|GGU66463 | 82% | 3e-23 | 59% | NM_205197.1 |
| 9 | *Gallus gallus* MMP16 mRNA for membrane type-matrix metalloproteinase 16, complete cds | 82% | 3e-23 | 59% | AB260848.1 |
| 10 | PREDICTED: *Meleagris gallopavo* matrix metallopeptidase 16 (membrane-inserted) (MMP16), mRNA | 82% | 3e-23 | 59% | XM_003205155.1 |

**Supplementary Table S1**. tBLASTn search results using a partial protein sequence encoded by *Biomphalaria glabrata* matrix metalloproteinase 1 (BgMMP1) sequence against the NCBI nucleotide database.
