# Supplementary material for: Excreted/secreted Schistosoma mansoni venom allergen-like 9 (SmVAL9) modulates host extracellular matrix remodelling gene expression
Source: Int J Parasitol. 2014 Jul;44(8):551–63. doi: 10.1016/j.ijpara.2014.04.002 (PMC4079936; doi:10.1016/j.ijpara.2014.04.002)
Supplement: Supplementary Table S2 — Quantitative reverse transcription (qRT)-PCR primers used in this study. [file mmc3.doc]

| **Gene** | **GenBank Accession No.** | **Forward (5’-3’)** | **Reverse (5’-3’)** | **Amplicon Size (bp)** | **Tm. (°C)** |
| --- | --- | --- | --- | --- | --- |
| *HPRT* | NM_013556.2 | CAG GCC AGA CTT TGT TGG AT | TTG CGC TCA TCT TAG GCT TT | 147 | 62 |
| *MMP2* | NM_008610.2 | GGA CCC CGG TTT CCC TAA GC | AGC TAT GAC CAC CAC CCT GC | 95 | 64 |
| *MMP9* | NM_013599.2 | CTG GAC AGC CAG ACA CTA AAG | CTC GCG GCA AGT CTT CAG AG | 145 | 64 |
| *MMP12* | NM_008605.3 | GAG TCC AGC CAC CAA CAT TAC | GCG AAG TGG GTC AAA GAC AG | 232 | 64 |
| *MMP13* | NM_008607.2 | CAG GGG AGA GCT TAG TTC TGT GA | GGT TCC AGC CAC GCA TAG TC | 85 | 60 |
| *MMP14* | NM_008608.3 | CTG CCC ACG GAC AAG ATC GA | ACC GGT AGT ACT TAT TGC CCC G | 85 | 60 |
| *MMP28* | NM_080453.2 | ACG CCA TCA GAG AGT TCC AGT | AGT ATC CGC AAC CCC ACA GC | 107 | 64 |
| *TIMP1* | NM_001044384.1 | GGG TGT GCA CAG TGT TTC CC | AGC AAA GTG ACG GCT CTG GT | 119 | 64 |
| *TIMP2* | NM_011594.3 | GGG CTG TGA GTG CAA GAT CA | GAC CCA GTC CAT CCA GAG GC | 85 | 60 |

**Supplementary Table S2.** Quantitative reverse transcription (qRT)-PCR primers used in this study.
